# A Laplacian-based framework for finite element human body model positioning

**DOI:** 10.3389/fbioe.2025.1599010

**Published:** 2025-08-29

**Authors:** Siyuan Chen, Xiaogai Li

**Affiliations:** Division of Neuronic Engineering, Department of Biomedical Engineering and Health Systems, KTH Royal Institute of Technology, Huddinge, Sweden

**Keywords:** human body model, positioning, Laplacian transformation, radial basis functions interpolation, traffic safety

## Abstract

Finite element human body model (HBM) positioning remains a challenge and automatic methods are essential to enable their effective use in a wide range of applications such as injury analysis in traffic accidents, sports, and forensic reconstructions. In this study, we present a new HBM positioning framework based on a hard-constrained Laplacian mesh deformation as its core, accompanied by both pre- and post-processing to enhance mesh quality, especially in joint areas, which are often a major source of mesh distortion during positioning. Specifically, the proposed pipeline leverages Blender to generate skin and skeleton surface meshes as target postures. The internal free node positions of the HBMs are then computed via Laplacian-based transformations with hard constraints. Notably, we propose the integration of thin-plate spline radial basis functions (RBFs) as an essential component of the framework to predict the positions of additional constraint nodes and to automatically repair distorted elements following Laplacian transformation during the pre and post processing steps. The performance of the framework was demonstrated through three cases using two HBMs (THUMS and PIPER), which involved substantial posture changes, including transitions from the seated to the standing position. Results show that the proposed framework yields smooth deformations while effectively minimizing mesh distortion. In particular, the inclusion of extra constraints effectively mitigates contact penetration and preserves anatomical fidelity, particularly in regions affected by large joint deformations or involving anatomically adjacent but physically unconnected components. In summary, this framework provides a robust and versatile solution for HBM positioning, offering a new alternative to existing approaches such as simulation-based and RBF interpolation-based methods.

## 1 Introduction

Finite element HBMs are mathematical representations of the human body that allow simulating biomechanical behavior under various scenarios, such as traffic accident analysis, sports biomechanics, and forensic case reconstruction (Östh et al., 2015; Li et al., 2019; Li and Kleiven, 2018; Bohman et al., 2022; Larsson et al., 2022; Boyle et al., 2020; Jakobsson et al., 2019; Boyle et al., 2019; Hu et al., 2019; Hwang et al., 2020; Tang et al., 2020; Pipkorn et al., 2021). Widely adopted HBMs in the field include Total Human Model for Safety (THUMS) (Iwamoto et al., 2002), Global Human Body Models Consortium (GHBMC) (Scott Gayzik et al., 2012), VIVA+ (John et al., 2022), SAFER HBM (Pipkorn et al., 2021), and PIPER (Beillas et al., 2016), each offering varying levels of anatomical detail and applications. As developing HBMs from scratch remains a highly time-consuming and complex task, they are usually developed with fixed geometry and posture.

For real-word application, HBMs need to be both personalized and properly positioned to align with the target scenario. In terms of personalization, our own work has proposed an efficient image-registration-based HBM technique for personalizing adult HBMs (Bengt Pipkorn Svein Kle et al., 2024; Li et al., 2023), a 2-month-old baby model (Chen et al., 2024), as well as subject-specific head model generation (Li, 2021). Additionally, other researchers have demonstrated the effectiveness of RBF-based morphing methods for HBM personalization (Larsson et al., 2022; Hwang et al., 2020; Tang et al., 2020; Hu et al., 2012; Li et al., 2011). A literature review by Hu et al. (2012) highlighted the capacities of thin-plate spline (TPS) RBFs in personalizing HBMs across diverse populations. Several studies have applied RBF-based methods to transform the baseline THUMS model and GHBMC model into various geometries representing different percentiles and body mass index (BMI) levels, demonstrating the strong potential of these methods in this field (Vavalle et al., 2014; Schoell et al., 2015; Shi et al., 2015). These techniques enable rapid HBM personalization within minutes. However, in contrast to personalization, HBM positioning remains a significant challenge, despite the development of several practical frameworks over the past decades.

Existing HBM positioning frameworks can be broadly categorized into simulation-based and interpolation-based pipelines. Simulation-based positioning pipelines leverage finite element solvers to iteratively adjust the model’s posture, often by applying forces or prescribed displacements. To the best of our knowledge, the earliest example of simulation-based positioning is the force-driven limb adjustment proposed by Parihar (2004). Later, Poulard et al. (2015) proposed a more systematic marionette approach that applies prescribed displacements incrementally using pulling cables to guide the model toward the target position. This method has been implemented in commercial software Oasys Primer (Bengt Pipkorn Svein Kle et al., 2024; Leledakis et al., 2021; Mohamed and Newlands, 2021; Östh and Bohman, 2020). Another notable simulation-based method is lightweight physics-based positioning approach implemented in the PIPER positioning framework (Beillas et al., 2015), based on the open-source SOFA framework (Faure et al., 2012). These simulation-based methods are capable of handling contact interactions and joint constraints, making them valuable and widely used approach for positioning HBMs, e.g., positioning the SAFER model (Bengt Pipkorn Svein Kle et al., 2024; Eliasson and Jacob, 2015; Maier et al., 2022), THUMS model (Leo et al., 2020),and GHBMC model (Corrales and Cronin, 2025). However, their reliance on finite element simulations makes them computationally expensive, particularly for detailed HBMs with millions of elements, which limits their applications requiring rapid repositioning. Although the PIPER framework does not require an FE solver, its ability to handle extreme postures is limited and can sometimes lead to instability issues.

Another important direction in this field is the development of interpolation-based positioning pipelines, which avoid the computational burden of simulations. Bucki et al. (2010) presented an elastic-registration-based method that combines hierarchical grid refinement with mesh repair to improve the quality of mapping results. However, this approach does not distinguish between human bones and soft tissues. In contrast, many methods treat bones and soft tissues separately. For instance, Jani et al. (2009) consider human bones as rigid bodies and apply affine transformations to achieve realistic joint motion, followed by isometric morphing to reposition the surrounding soft tissue nodes. Additionally, various interpolation functions for deformable parts, including dual kriging interpolation (De et al., 2012), contour-based positioning approaches (Sudipto Mukherjee Rahul Goyal Nataraju Vusirikala Dhaval Jani et al., 2012; Chhabra et al., 2024), as well as landmark-based radial basis function (RBF) interpolation, have also been developed (Li et al., 2011; Grassi et al., 2011). Hwang et al. (2016) presented a comprehensive theoretical framework for RBF interpolation, and applied it specifically to the repositioning of HBMs. After a decade of development, RBF-based techniques have evolved into a widely adopted HBM positioning pipeline, which offer improved computational efficiency compared to simulation-based pipelines (Hu et al., 2019; Tang et al., 2020; John et al., 2022; Yuan et al., 2023; Zhang et al., 2017; Phillip, 2018). To improve the efficiency and accuracy of RBF-based pose transformations, some studies have introduced modular approaches that divide the body into different regions and apply transformations sequentially (John et al., 2022; Zhang et al., 2017). Furthermore, workflows incorporating techniques such as iterative subsampling and spatial partitioning have been proposed to enhance computational efficiency (Janák et al., 2020).

Laplacian-based transformations have become widely used for mesh editing in classical computer graphics due to their ability to preserve local geometric structures while accommodating global shape changes (Sorkine-Hornung, , 2005; Zhou et al., 2005). The fundamental idea behind Laplacian deformation is to encode shape changes as local differences between each vertex and its neighbors, allowing for the preservation of fine geometric features during smooth mesh deformation. One key advantage is computational efficiency, as the transformation often leads to a sparse linear system that can be solved efficiently. Additionally, by leveraging the inherent topology of the original mesh, these methods facilitate smooth deformations through the even distribution of displacements across the mesh. Therefore, Laplacian transformations have been extensively applied in general mesh editing tasks due to their efficiency and ability to preserve local geometric features (Sorkine et al., 2004; Sorkine and Alexa, 2007). However, Laplacian transformations do not explicitly control internal spatial relationships. As a result, most of their applications have been limited to surface meshes (Sorkine-Hornung, , 2005; Zhou et al., 2005; Sorkine et al., 2004; Sorkine and Alexa, 2007; Alexa et al., 2007; Dong et al., 2023), and they have not yet been applied for HBM positioning. Its application to HBM positioning presents unique challenges, including maintaining internal anatomical structures, preserving element quality, and handling regions with large joint rotations, which are not trivial extensions from surface-based Laplacian deformation.

This study aims to address above challenges and develop a robust HBM positioning framework based on a hard-constrained Laplacian transformation, offering a new alternative beyond current simulation-based and RBF-based approaches in this field. The target postures of skin and skeleton surfaces are generated using rigging and skinning techniques, which serve as boundary constraints for the subsequent mesh deformation process. We further introduce a preprocessing step to prevent contact penetration and ensure anatomical plausibility, followed by a postprocessing step to enhance element quality after positioning. The paper is organized as follows: first, the overall workflow of the proposed framework is presented; this is followed by introduction of the employed HBMs and three different positioning cases, along with a theoretical background of the Laplacian transformation and practical implementation of each step in the pipeline; in the results section, the positioning results of the three cases are presented, followed by a evaluation of mesh quality and computation time; finally, the advantages and limitations of the proposed framework are discussed.

## 2 Methods

**FIGURE 1:**
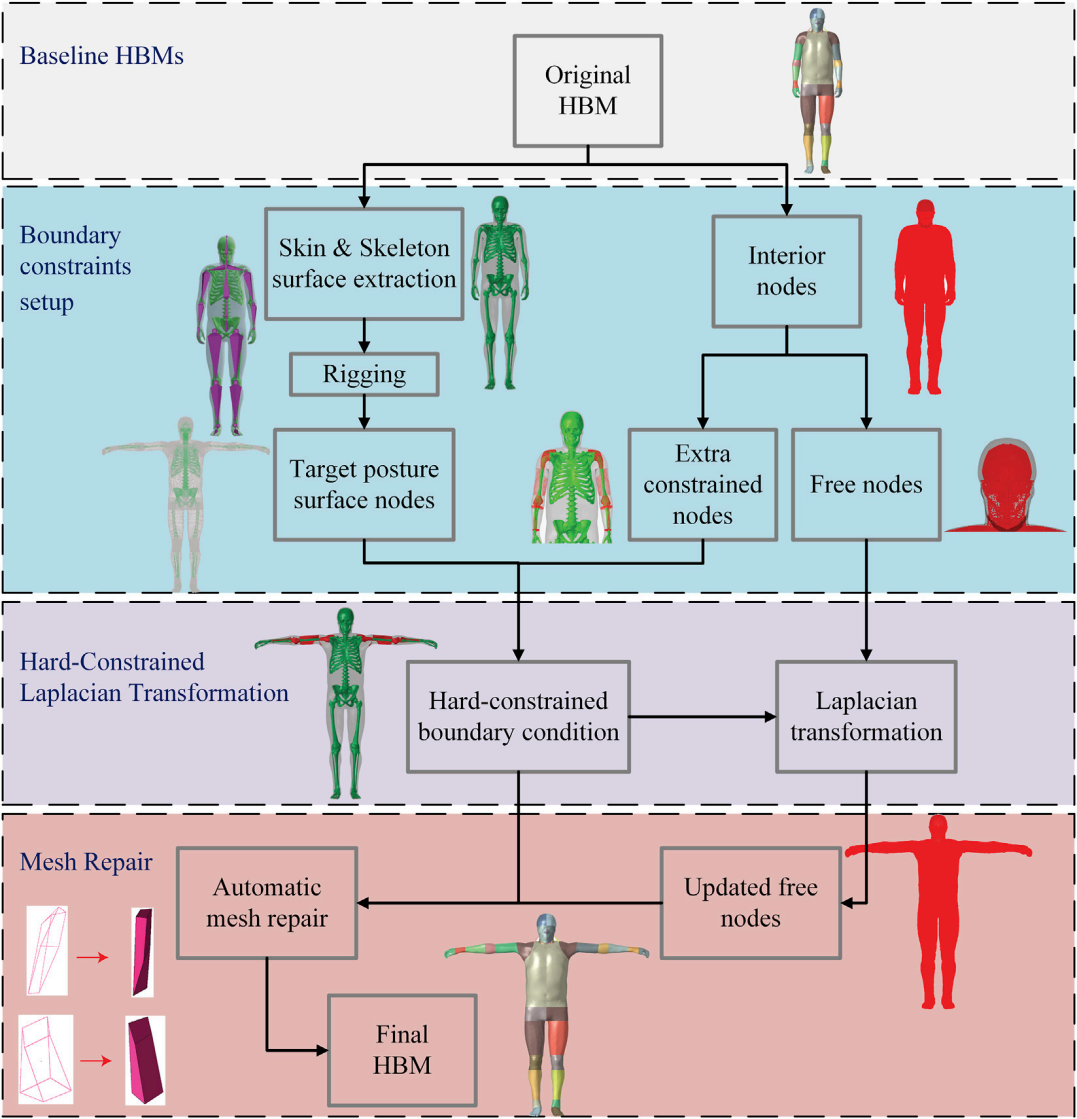
Flowchart of the proposed positioning framework, comprising three main steps: Pre-processing step for boundary constraints setup, Hard-constrained Laplacian transformation and Post-processing for mesh repair. A representative positioning case is presented to illustrate each step of the framework. These stages correspond to Sections 2.2 (Pre-processing), Section 2.3 (Laplacian Transformation), and Section 2.4 (Post-processing), respectively. The baseline HBMs used in this framework are introduced in Section 2.1.

This section presents the workflow of the proposed HBM positioning framework based on Laplacian transformation. The process consists of three steps: pre-processing for setting up boundary constraints, which then feed into the hard-constrained Laplacian transformation, and finally post-processing mesh repair to improve element quality, as shown in Figure 1.

### 2.1 Baseline HBMs and evaluation cases

**FIGURE 2:**
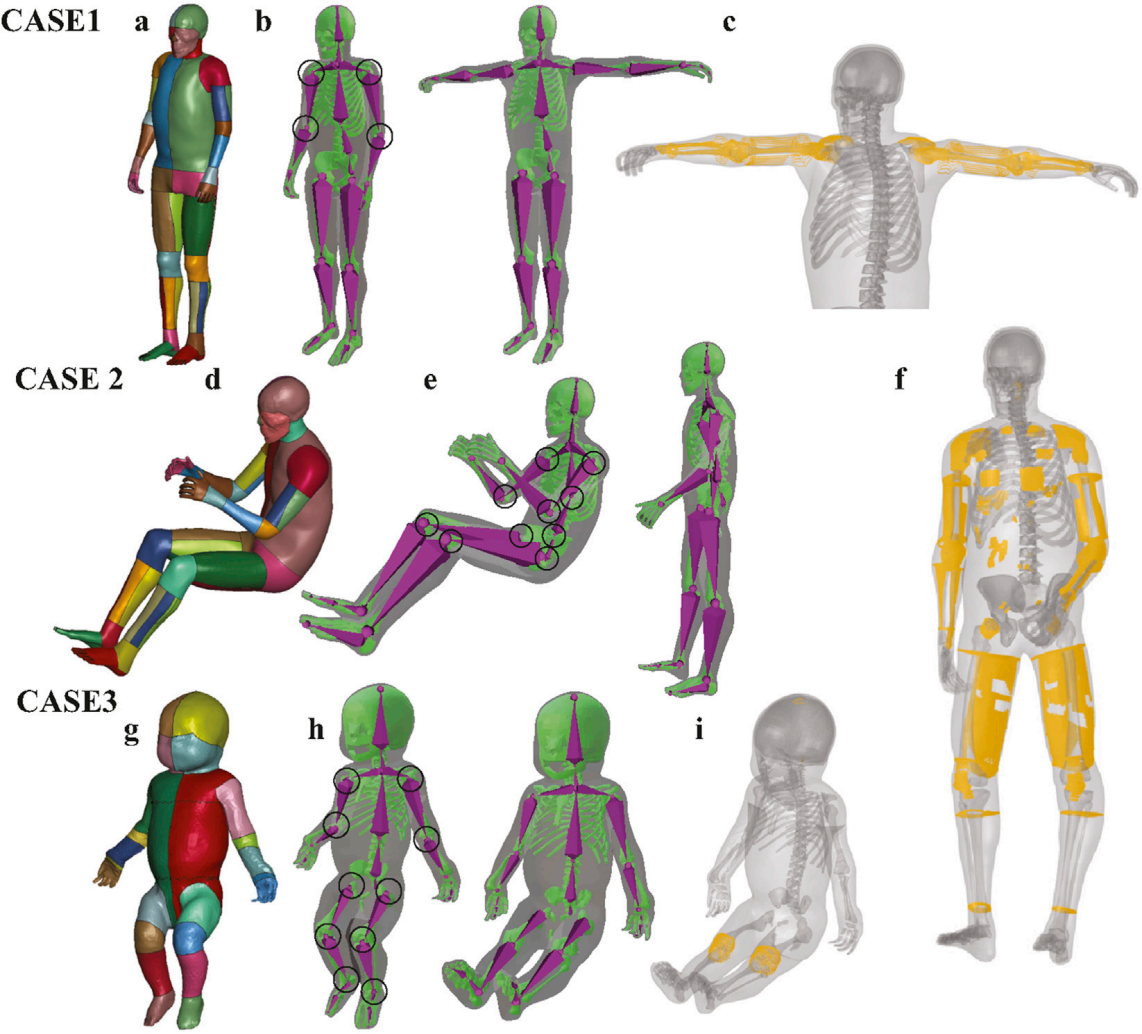
Demonstration of boundary constraint node generation in three representative cases. The boundary constraint nodes consist of two components: (1) surface nodes of the skin and skeleton, and (2) joint-related nodes near the transformed joints. The skin and skeleton surface nodes were first extracted from the baseline HBM and subsequently repositioned to the target posture using Blender with armature binding and skinning to control joint rotations. **(a,d,g)** Show the original HBMs used in this study, including the THUMS pedestrian baseline model, the occupant baseline model, and the PIPER infant model. **(b,e,h)** Illustrate the generation of surface constraint nodes for Cases 1, 2, and 3, where the skin surface is visualized in transparent gray, the skeleton surface in green, and the armature in purple. The joints that underwent rotational adjustment are highlighted with black circles. **(c,f,i)** Show the additional constrained nodes (yellow) in Cases 1, 2 and 3. These nodes were generated via RBF interpolation between corresponding surface nodes of the baseline and target postures. For clarity, the skin and skeleton are displayed transparently to better highlight the spatial distribution of the extra constraint nodes.

To evaluate the proposed framework, we applied it to the 50th percentile male THUMS model (version 4) (Iwamoto et al., 2002) in two cases, using the pedestrian and occupant configurations as the original models (Figures 2a,d). The THUMS model is a highly detailed HBM developed by Toyota and its partners, consisting of approximately 2 million elements and 0.77 million nodes (Iwamoto et al., 2002). To further demonstrate the versatility of the positioning framework, we also applied it to the PIPER infant model (as shown in Figure 2g), which was morphed from the 1.5-year-old PIPER child model, with a total of 0.54 million elements and 0.14 million nodes (Chen et al., 2024). These models serve as the baseline HBMs in the beginning of the proposed framework (Figure 1).

A total of three cases are used to evaluate the framework in this study. In the first case, the pedestrian THUMS model was adjusted to a T-pose, where the arms are fully extended horizontally to the sides at shoulder level and the lower body remaining in the original pedestrian stance. The joints involved in this transformation mainly include the shoulders and elbows (Figure 2b). In the second case, the occupant THUMS model was repositioned to a pedestrian gait posture representing a mid-stride phase of walking. This transformation was achieved by adjusting the following joints: shoulders, elbows, thoracic spine, lumbar spine, hips, and knees (Figure 2e). In the last case, the original PIPER infant model, which was initially in a lifted posture, was repositioned to a seated occupant posture adapted to fit an infant safety seat. In this posture, the torso leans slightly backward, the legs are bent at the hips and knees, and the arms rest naturally along the sides with the hands positioned near the lap (Figure 2h).

### 2.2 Boundary constraints defined by skin and skeleton surfaces, along with extra constrained nodes (pre-processing step)

The Laplacian transformation relies critically on the configuration of boundary nodes, which act as positional constraints during the computation of the positions of the remaining free nodes. In our proposed framework, the boundary node configuration comprises two primary components: (1) surface nodes of the skin and skeleton (Figures 2b,e,h), and (2) extra constrained nodes (Figures 2c,f,i). Further details of each step are introduced below.

#### 2.2.1 Skin and skeleton surface constraints

The skin and skeleton surface constraints are generated using Blender (version 3.6) (Blender Development Team, 2023), and are employed to define the overall target posture. In this step, both skin and skeleton surface models in the original HBMs were first extracted and converted into.obj files. An armature was then created through the rigging process to closely replicate the geometry of the original skeleton (Blender Development Team, 2023), followed by binding the armature to the skeleton and skin surfaces through a process called skinning. An automatic weight assignment was initially performed using built-in algorithms, followed by manual refinement of vertex weights via weight painting to ensure accurate skin and skeleton deformation in response to armature movements. All steps were performed using native functionality in Blender 3.6 without any external add-ons.

#### 2.2.2 Extra constraint nodes generation

The extra constrained nodes are generated using an RBF interpolation algorithm and serve as key constraints to prevent mesh penetration by providing localized control in areas of large joint deformation and in regions where anatomically adjacent components are not topologically connected (e.g., internal organs and the nearby skin or skeletal structures that are anatomically close but not connected in the mesh). The surface nodes extracted from Blender-defined skin and skeleton meshes effectively determine the target posture. However, large-angle joint transformations may lead to undesired mesh intersections, particularly near contact interfaces between anatomical structures. To address this, the proposed framework introduces an additional constraint handling step, which incorporates a set of extra constrained nodes located in regions prone to penetration as supplementary boundary constraints.

These extra constrained nodes are manually selected based on prior experience and *post hoc* observations of deformation results. Specifically, after an initial transformation is performed using only surface constraints, the user can identify problematic areas where element penetration or distortion occurs. It is generally sufficient to constrain only a subset of nodes within the penetrated region, since the positions of the remaining free nodes can be inferred through Laplacian transformation. In practice, the best strategy for selecting extra nodes is to target those located on or near the intersection interfaces where penetration is observed. These are typically found at transition zones between anatomically adjacent components, such as soft tissues near bones or internal organs adjacent to the skeletal structure or skin surface. Constraining these interface nodes helps propagate anatomically plausible deformation and effectively eliminate local mesh penetration. The target positions of the extra constrained nodes are estimated using a localized RBF-based interpolation method, which leverages its ability to capture fine-scale spatial relationships. The detailed procedure is as follows:

• A KDTree (a data structure for fast nearest-neighbor queries in multidimensional space) is constructed from the original nodes, including the skin, skeleton, and extra constrained nodes, to efficiently identify the k nearest neighbors of each extra constrained node.
• For each node, the corresponding positions of its k -nearest neighbors in the target posture are used to construct a local interpolation using a thin-plate spline (TPS) kernel.
• The target position of each node is then computed using the local TPS interpolation.

### 2.3 Hard-constrained Laplacian transformation

As part of the proposed positioning framework, a hard-constrained Laplacian transformation was applied to compute the positions of the remaining free nodes, based on the locations of the predefined boundary nodes (Section 2.2). Section 2.3.1 introduces the computation of discrete differential coordinates and the construction of the Laplacian matrix based on the topological structure of HBM. Section 2.3.2 describes the process of solving a linear system to determine the positions of interior free nodes, subject to the constraints defined by the known boundary nodes.

#### 2.3.1 Discretization of differential coordinates and Laplacian matrix construction

Let $M=\left(V,E\right)=\left(V,\left\{E_{1},E_{2},E_{3}\right\}\right)$ be a given HBM with n nodes. V denotes the set of vertices, referring to all nodes in the 3D mesh model. E denotes the set of elements, including the set of solid elements $E_{1}$ , the set of shell elements $E_{2}$ , and the set of 1D elements $E_{3}$ . The continuous form of Laplace–Beltrami operator is defined as shown in Equation 1 (Manfredo Perdigao Do Carmo and Francis, 1992):

$$\Delta f\left(p\right)=\text{div}\left(\text{grad}f\left(p\right)\right),$$ (1)

where Δf(p) represents the Laplacian of the function f at point p , grad(f(p)) denotes the gradient of f at point p , and div(⋅) is the divergence operator.

Considering that the original HBM M is a discrete approximation of a smooth surface combination, the appropriate discretization of the differential coordinate $\delta_{i}$ at vertex $v_{i}$ can be written as shown in Equation 2

$$\delta_{i}=\left(\delta_{i}^{\left(x\right)},\delta_{i}^{\left(y\right)},\delta_{i}^{\left(z\right)}\right)=\frac{1}{deg_{i}}\underset{j\in N\left(i\right)}{\sum }\left(v_{i}-v_{j}\right)w_{ij},$$ (2)

where $deg_{i}$ is the degree of vertex i , defined as the sum of the weights between vertex i and its neighbors ( $deg_{i}=\sum_{j\in N\left(i\right)}w_{ij}$ ), N(i) is the set of neighbors of vertex i , $v_{i}$ and $v_{j}$ represent the positions of vertices i and j , respectively, and $w_{ij}$ are the weights between the vertices i and j .

The differential coordinate encodes the local geometric details by representing the relative position of a vertex with respect to its immediate neighbors, capturing fine-scale shape features independent of global translation or rotation. In this study, the neighborhood N(i) is defined based on the mesh connectivity: two nodes are considered neighbors if they belong to the same element. This adjacency definition ensures that the resulting Laplacian matrix faithfully captures the full topological structure of the original HBM and preserves structural coherence during the deformation process. Additionally, we use inverse cubic relationship to define the weight $w_{ij}$ , meaning that as the distance increases, the weight rapidly attenuates

$$w_{ij}=\frac{1}{d_{ij}^{3}+\epsilon },$$

where $d_{ij}=\|v_{i}-v_{j}\|$ is the Euclidean distance between vertices $v_{i}$ and $v_{j}$ , and ϵ is a small positive constant added to avoid division by zero.

Let A be the weighted adjacency matrix of the mesh:

$$A_{ij}=\left\{\begin{array}{cc} w_{ij},\; & \left(i,j\right)\in E,\\ 0,\; & \text{otherwise}, \end{array}\right.$$

And let D be the diagonal matrix such that:

$$D_{ii}=deg_{i}=\underset{j\in N\left(i\right)}{\sum }w_{ij}.$$

The Laplacian matrix L is defined as:

L=D−A.

The transformation of the vector of Cartesian coordinates to the vector of differential coordinates can be represented in matrix form as Equation 3:

δ=LV, (3)

where L is the Laplacian matrix and V is the vector of Cartesian coordinates of the vertices.

#### 2.3.2 Solving constrained linear system

The Laplacian-based deformation assumes that the differential coordinates of free nodes remain consistent between the original and target postures. To restore the global coordinates in the target posture from the preserved differential coordinates δ , a linear system involving the Laplacian matrix L has to be solved. The present hard-constrained Laplacian transformation enforces the following constraints:

$$v_{j}=c_{j},\;j\in C,$$

where $c_{j}$ is the known Cartesian coordinate of vertex $v_{j}$ , and C is the set of constrained nodes as defined in Section 2.2.

The system of equations for the hard-constrained Laplacian transformation can be as Equation 4:

$$\left(\begin{array}{cc} L_{FF} & L_{FC}\\ L_{CF} & L_{CC} \end{array}\right)\left(\begin{array}{c} v_{\text{free}}\\ c_{\text{fixed}} \end{array}\right)=\left(\begin{array}{c} \delta_{\text{free}}\\ \delta_{\text{fixed}} \end{array}\right),$$ (4)

where $L_{FF}$ and $L_{FC}$ are submatrices of the Laplacian matrix L , $v_{\text{free}}$ represents the positions of the free vertices, and $c_{\text{fixed}}$ represents the known positions of the constrained vertices. By rearranging, the equation for the free vertices becomes Equation 5:

$$L_{FF}v_{\text{free}}=\delta_{\text{free}}-L_{FC}c_{\text{fixed}}.$$ (5)

where $\delta_{\text{free}}$ is computed from the original mesh based on the Equation 2.

In our framework, the resulting sparse linear system is solved using the standard conjugate method, as formulated in Equation 6.

$$v_{\text{free}}=\arg\underset{v_{\text{free}}}{\min}{\left\|L_{FF}v_{\text{free}}-\left(\delta_{\text{free}}-L_{FC}c_{\text{fixed}}\right)\right\|}^{2}.$$ (6)

### 2.4 Mesh quality improvement (post-processing step)

Similar to the joint constraint handling process, RBF interpolation is employed to automatically repair “distorted elements” with a Jacobian value less than 0.3. In the first step of the repair process, the distorted elements and their neighboring elements are extracted, where nodes belonging to distorted elements are classified as defective nodes, and all remaining nodes in these elements are categorized as reference nodes. Subsequently, the original model, assumed to be a well-conditioned baseline model, is used to establish spatial relationships between defective nodes and reference nodes. These relationships serve as a foundation for corrective adjustments. In the final step, the relative positions of defective nodes with respect to reference nodes in the original model are mapped onto the positioned model. Based on the updated positions of reference nodes in the positioned model, RBF-based interpolation is applied to automatically adjust the locations of defective nodes accordingly.

The Jacobian value and aspect ratio are commonly used metrics for assessing mesh quality, where the Jacobian value reflects the local transformation from the reference element to the standard element configuration, and the aspect ratio measures the relative stretching of an element, with high values suggesting elongated or skewed shapes. In this study, we evaluate mesh quality by counting the number of elements with a Jacobian value below 0.3, negative volume elements (J<0) , and elements with an aspect ratio greater than 10. Negative volume elements with J<0 indicate totally inverted elements, which are physically invalid and not generally permissible in finite element simulations. The thresholds of 0.3 for the Jacobian value and 10 for the aspect ratio are based on empirical practice and are used to quantify the number of distorted mesh elements in FE models.

## 3 Results

**FIGURE 3:**
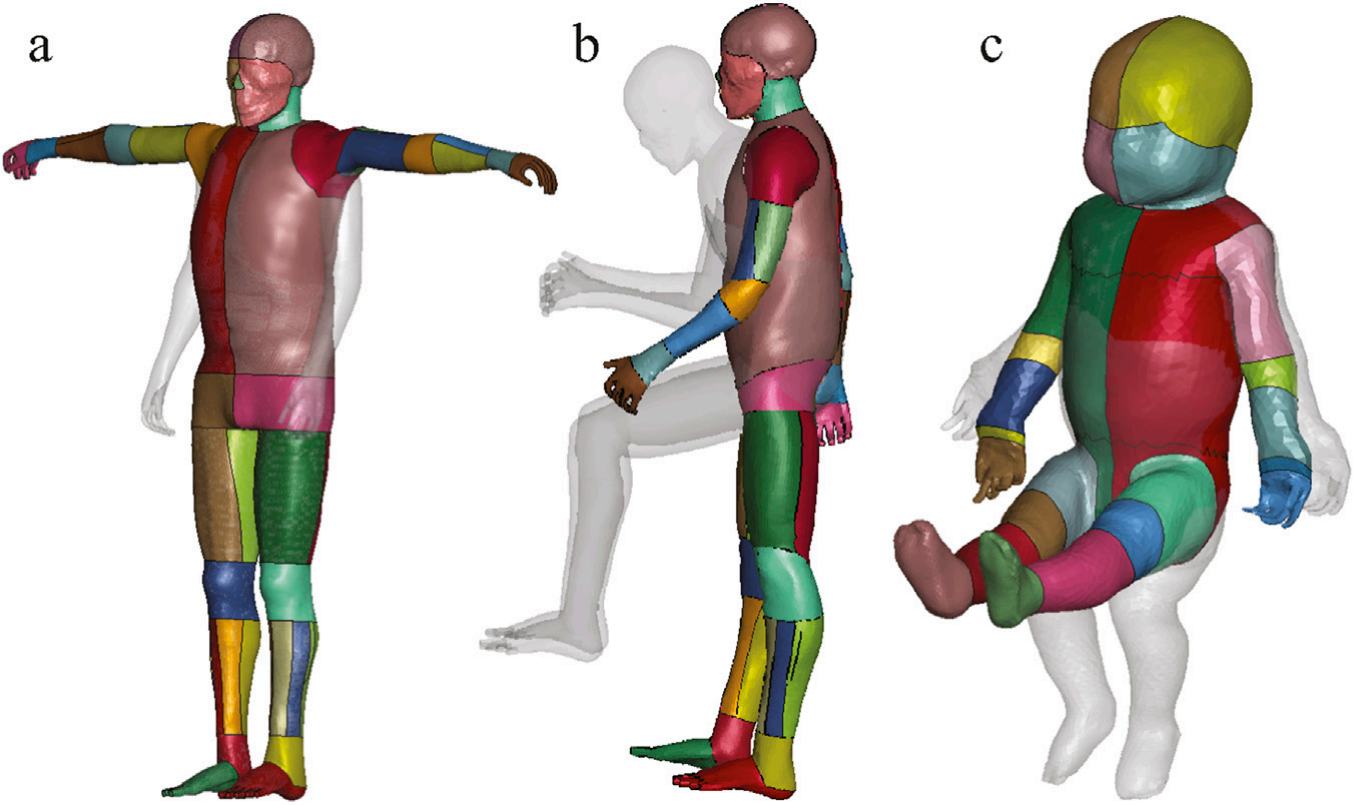
The positioned HBMs combined with their transparent original position in gray, demonstrating correct anatomical alignment and no visible self-intersections. **(a)** Case 1: the pedestrian version of the THUMS model was adjusted to a T-pose. **(b)** Case 2: the occupant version of the THUMS model was repositioned to a gait posture. **(c)** Case 3: the lifted PIPER infant model was adjusted to a seated position.

The positioned HBMs in the prescribed postures for all three cases are shown in Figure 3. All positioned models were successfully transformed into the target postures with smooth surface transitions, sufficient mesh quality, and anatomically reasonable configurations, making them ready for subsequent finite element simulations. Table 1 indicates that the mesh quality degraded after the Laplacian transformation in all three cases due to the large joint displacements involved. Among three cases, Case 2 exhibited the most severe degradation, as it involved the highest number of joint transformations and the largest posture change (from an occupant position to a walking posture). In contrast, Case 3, despite involving a large posture change, was less affected because the PIPER model has a lower mesh density and fewer anatomical components compared to THUMS. After applying the proposed automated mesh repair process, a substantial portion of the distorted elements were successfully corrected, with approximately 80% of the elements with J<0.3 repaired in Cases 1 and 2, and the mesh quality in Case 3 restored to the same level as the original model. Moreover, Table 1 reports the number of negative volume elements, which are of particular concern for finite element simulations. Notably, for Cases 1 and 3, no negative volume elements remained after the automatic repair step, while in Case 2, 89% of the negative volume elements were eliminated, with only 12 elements left, which significantly reduces the need for further manual mesh correction, especially considering the model contains approximately 77,000 nodes.

**TABLE 1:** Comparison of element quality before and after applying the Laplacian transformation and mesh repair postprocess across the three evaluated cases.

Condition	CASE 1	CASE 2	CASE 3
Number of Elements with J<0.3			
Original	10	10	48
After Laplacian	167	469	93
After Repair	25	95	48
Number of Elements with J<0			
Original	0	0	0
After Laplacian	39	114	1
After Repair	0	12	0
Number of Elements with Aspect Ratio > 10			
Original	186	176	152
After Laplacian	216	280	184
After Repair	218	262	185
CPU Execution Time of Laplacian Transformation (s)			
After Laplacian	216.3	2038.0	33.1
GPU Execution Time of Laplacian Transformation (s)			
After Laplacian	37.3	145.0	9.2

Element quality is assessed using the number of elements with Jacobian value J<0.3 , negative volume, and aspect ratio >10 . Both CPU and GPU execution times are also reported to illustrate the computational performance of the Laplacian transformation.

The CPU execution times for solving the Laplacian system are also provided in Table 1, demonstrating the computational efficiency of the proposed method. Additionally, GPU execution times for the positioning process are reported to highlight the significant performance improvements enabled by hardware acceleration. Among the three cases, Case 2 required the longest computation time of approximately 34 min on the CPU and 2 min on the GPU. In contrast, Case 3 completed in only 33 s on the CPU. Case 1 involved fewer joint transformations, which led to faster convergence of the Laplacian solver, with a CPU execution time of 216 s in this study. The computations in this study were performed on a regular workstation equipped with an Intel Xeon W-2223 CPU @ 3.60 GHz and an NVIDIA Quadro P1000 GPU.

## 4 Discussion

This study presents a new, efficient, and robust HBM positioning framework based on Laplacian transformation, which enables accurate posture adaptation of high-resolution HBMs across diverse scenarios while offering a practical alternative to existing positioning pipelines such as simulation-based deformation and modular RBF interpolation. The primary contribution of this framework lies in the integration of Laplacian-based deformation with a practical implementation pipeline for quality element generation with minimal manual work. Specifically, we leverage Blender to generate pose-specific skin and skeleton surfaces, apply a Laplacian transformation to drive the mesh deformation, and employ RBF interpolation to automatically repair local distortions and improve mesh quality. Its effectiveness is validated through three representative cases involving large, multi-joint transformations, such as occupant-to-pedestrian and standing-to-seated transitions. Furthermore, the transformed models exhibit significantly improved mesh quality, minimizing the need for further manual mesh correction.

### 4.1 Why and how extra constrained nodes are introduced prior to Laplacian transformation?

**FIGURE 4:**
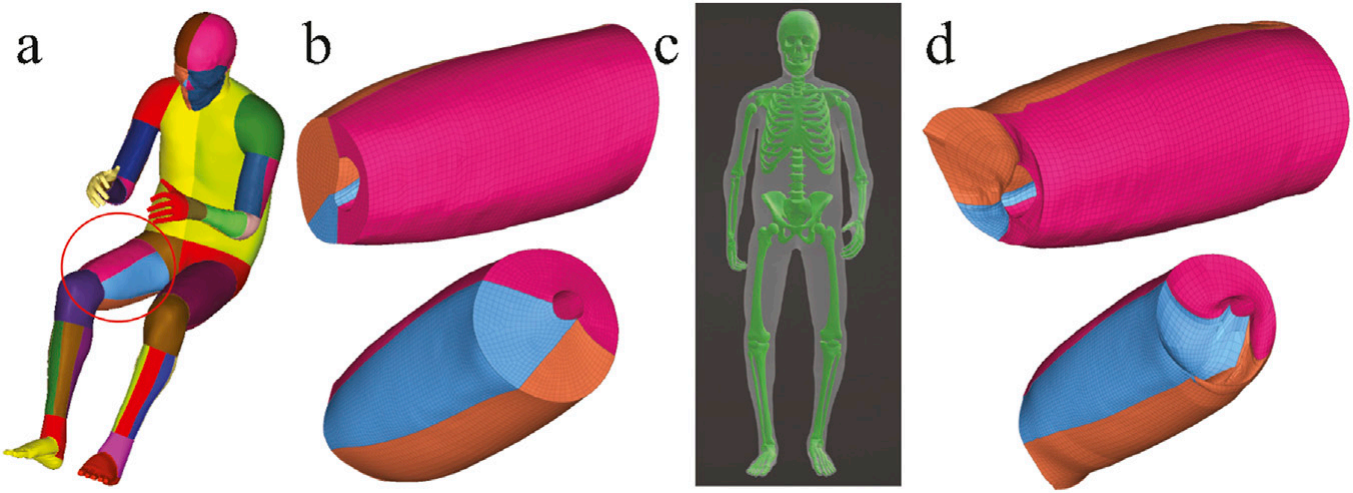
Illustration using the right thigh in Case 2, showing only the solid elements of the HBM for better structural visualization. **(a)** Segmented HBM with the thigh region of interest circled. **(b)** Mesh quality of the right thigh in the original model, showing smooth end planes. **(c)** Skin and skeleton surface constraints generated in Blender define the target posture, but lack constrained nodes near joint regions. **(d)** Resulting thigh mesh after Laplacian transformation without extra constraints, showing loss of smoothness on end planes, with visible mesh distortions and interpenetration.

Laplacian transformations are well-known for their ability to preserve local mesh topology, their direct application to high-resolution HBMs presents unique challenges due to large joint articulations, complex anatomical structures, and intricate contact interfaces. A core assumption in Laplacian-based deformation (as shown in Equation 2) is that the differential coordinates, which represents local geometric differences between a node and its neighbors, remains consistent before and after deformation. However, when the posture of an HBM changes, particularly for those nodes in regions near joints, this assumption will not hold. In high-resolution HBMs, large joint rotations can induce highly nonlinear changes in the local geometry, leading to significant discrepancies between the original and deformed differential coordinates. Due to this inherent limitation, additional constraints are required to preserve anatomical accuracy and maintain mesh quality during Laplacian-based HBM positioning. Without additional constraints, these discrepancies may propagate through the system, resulting in element distortions even in regions distant from the joints, as shown in Figure 4. The introduction of extra constrained nodes allows the deformation to better enhance anatomical plausibility, reflecting the anatomical reality of human posture adjustment (e.g., altering the hip or knee joint posture primarily affects the boundaries of the thigh, without significantly changing its internal structure).

**FIGURE 5:**
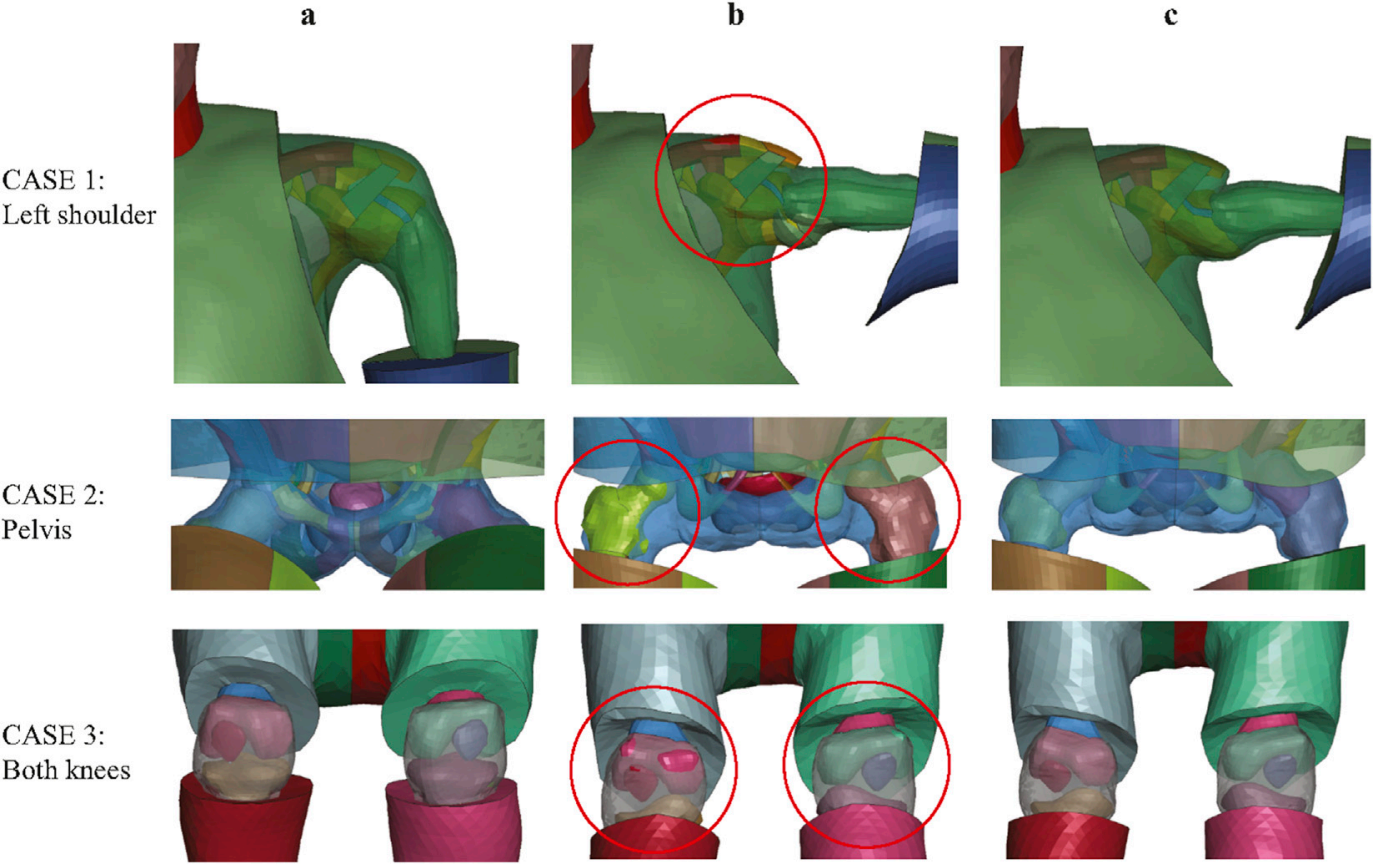
Significance of joint-related constraints in avoiding mesh penetration across three cases. **(a)** Original model; **(b)** results without joint-related constraints showing mesh penetrations (red circles); **(c)** results with joint-related constraints applied.

Another important motivation for introducing additional constraints is that Laplacian-based deformation cannot account for spatial relationships between anatomically adjacent but topologically disconnected structures. Figure 5 compares contact penetration in joint regions with and without the inclusion of additional joint-related constraint nodes. All three cases demonstrate that relying solely on surface constraints from the target skin and skeleton leads to inevitable contact penetrations near joints. This limitation also arises in regions where the local geometry of individual components remains unchanged. For instance, it is necessary to constrain specific nodes on the pectoral in Case 2 and the brain in Case 3, respectively, as shown in Figure 2. These anatomical components do not share direct node connectivity with adjacent structures, and therefore additional constrained nodes must be predefined to avoid penetration during deformation. It is worth noting that the number of extra constraint patch nodes can be large in some cases. This is, however, necessary, especially in cases involving substantial joint rotations or anatomically adjacent yet topologically disconnected structures. The selection of these nodes is based on empirical knowledge. For users without prior experience, it is often necessary to first perform an initial HBM transformation with only skin and skeleton surface nodes to identify where additional constraints are required, guided by observed mesh penetration and simple mesh quality criteria (e.g., J<0.3 ). However, the IDs of the extra nodes can be stored for reuse, thereby reducing the processing time in subsequent workflows.

### 4.2 Difference between current HBM positioning pipelines

Existing HBM positioning methods, including both simulation-based and interpolation-based pipelines, are already well developed and have made significant contributions to the field of biomechanics (Hu et al., 2019; Tang et al., 2020; John et al., 2022; Poulard et al., 2015; Leledakis et al., 2021; Östh and Bohman, 2020; Zhang et al., 2017; Phillip, 2018). The key advantage of the simulation-based pipeline is that it leverages commercial software such as Primer and LS-DYNA, enabling relatively straightforward positioning setup while incorporating contact definitions to help mitigate mesh penetration at contact interfaces. Our previous study positioned the SAFER occupant model to a pedestrian posture using the simulation-based approach (Bengt Pipkorn Svein Kle et al., 2024). The quality of the resulting HBM is sensitive to the simulation setup, particularly the contact definitions and prescribed boundary input parameters, and for some cases of significant posture variation, it is necessary to divide the process into multiple intermediate postures to ensure acceptable mesh quality (Bengt Pipkorn Svein Kle et al., 2024). Another limitation of this method is it needs long simulation time for detailed HBM. For the SAFER HBM of approximately 0.41 million elements, each positioning simulation requires about 2 hours on a high-performance computing cluster with 256 CPU cores, assuming no premature termination or solver errors occur during the simulation.

A key challenge for non-simulation-based methods lies in determining the positions of the remaining nodes based on the locations of control points. In contrast to Laplacian-based transformation, interpolation-based approaches determine the position of each free node based on its spatial proximity to selected control points, rather than the neighbor nodes share the same elements. Global interpolation can lead to unrealistic node positions under large pose changes due to abrupt displacements of the reference points because it can not explicitly preserve local geometric features (Eiderbäck and Jahnke, 2023). This limitation is particularly evident in complex posture transformations. For example, as shown in Supplementary Table SA2, under the same boundary conditions, global RBF interpolation led to substantial mesh degradation in Case 2, with more than 10,000 elements exhibiting Jacobian values J<0.3 , versus 469 distorted elements by Laplacian transformation. However, the current popular modular (block-wise) interpolation-based methods effectively overcome the limitations of global interpolation by partitioning the HBM into anatomically meaningful components and restricting interpolation to local regions rather than the entire model (John et al., 2022; Zhang et al., 2017). Such modular strategies have been shown to improve both computational efficiency and mesh quality, and have proven effective in both the personalization and positioning of multiple HBMs in many previous studies (Hu et al., 2019; Tang et al., 2020; John et al., 2022; Yuan et al., 2023; Zhang et al., 2017; Phillip, 2018). To overcome the limitations of global RBF interpolation in HBM positioning, our framework adopts a different strategy by constructing a Laplacian matrix based on the topological connectivity of HBM. This topology-driven deformation ensures consistent behavior across the mesh and inherently preserves local geometric features, without requiring explicit spatial neighbor searches. Moreover, the transformation process is governed by solving a sparse Laplacian system, which ensures computational efficiency while simultaneously determining the positions of all remaining nodes.

### 4.3 Mesh quality assurance by both pre and post-processing

Maintaining high mesh quality and ensuring accurate contact definitions after positioning are critical for achieving stable and reliable finite element simulations. One primary challenge in HBM transformation is maintaining the correct separation between contacting surfaces, such as bones, muscles, and organs. Different transformation methods may introduce new intersections, leading to unintended mesh penetration between different anatomical structures. Simulation-based positioning approaches, which apply physically motivated constraints and contact definitions during the transformation process, can inherently reduce mesh penetration and better preserve contact integrity, particularly in complex joint regions (Mohamed and Newlands, 2021; Eliasson and Jacob, 2015). Tang et al. (2023), referencing kinesiology and the anatomy near joints, proposed a novel and efficient morph-contact algorithm to address penetration issues around joint regions. To address the inherent limitations of Laplacian-based deformation, as discussed in Section 4.1, we propose a novel integration of localized RBF interpolation applied prior to the transformation process, in contrast to previous *post hoc* mesh repair methods to correct contact penetrations. Leveraging its strength in capturing spatial relationships, we proactively constraints some extra nodes to guide the further Laplacian deformation, which effectively prevents penetration in high-deformation regions and ensures the preservation of anatomical integrity, especially around joints.

Both posture adjustments and morphological modifications in HBMs may lead to degradation in element quality, particularly in regions with large deformations. Mesh smoothing techniques have been widely used to improve the shape of irregular elements (Sudipto Mukherjee Rahul Goyal Nataraju Vusirikala Dhaval Jani et al., 2012; Tang et al., 2023; Christoph Klein et al., 2021). In our framework, since the Laplacian transformation is based on the topology of the original model, it cannot adaptively repair distorted mesh elements, especially under the large geometric discrepancy between the source and target models. To address this challenge, similar to the preprocessing step, we do not treat RBF as a general transformation tool. Instead, the post-processing repair step specifically targets defective nodes by leveraging their relative spatial relationships with neighboring nodes in the original model. This approach significantly reduces the need for manual mesh correction and is fully compatible with automated, programmable implementation within the positioning pipeline. In our three cases, the repositioned models after automatic mesh repair can be used directly for finite element simulation without requiring additional manual mesh correction, aside from a small number of distorted elements in Case 2, highlighting the practicality of the proposed pipeline in minimizing manual post-processing efforts.

### 4.4 Limitations and future directions

Several limitations of the proposed framework warrant further investigation. First, the posture rigging process in Blender requires considerable manual intervention. While this setup is a one-time effort for each baseline model, it remains a bottleneck for fully automated workflows. Second, the placement of extra constrained nodes currently relies on empirical experience and *post hoc* analysis of deformation outcomes. Future work could explore automated strategies for constraint placement, guided by target posture geometry and anatomical landmarks, to improve robustness and reduce user intervention. Finally, the current implementation uses a uniform Laplacian weighting scheme without considering more advanced formulations. Future research could investigate anisotropic weighting or soft-constrained Laplacian methods to improve deformation accuracy and reduce dependency on extra constraints near joints.

## 5 Conclusion

This study provides a new, simulation-free HBM positioning framework based on a hard-constrained Laplacian transformation. The framework integrates dedicated preprocessing and postprocessing steps to improve mesh quality and anatomical plausibility. Its effectiveness has been successfully demonstrated across multiple cases, including extreme posture changes, highlighting its robustness, versatility, and capacity to handle complex, multi-joint transformations, especially for highly detailed HBMs. Furthermore, the preprocessing and postprocessing steps are generalizable and can be readily incorporated into other HBM positioning pipelines, offering practical value for improving mesh quality and reducing the need for manual correction in downstream finite element simulations.

## Data Availability

The original contributions presented in the study are included in the article/Supplementary Material, further inquiries can be directed to the corresponding author.
