# Supplementary material for: A Laplacian-based framework for finite element human body model positioning
Source: Front Bioeng Biotechnol. 2025 Aug 29;13:1599010. doi: 10.3389/fbioe.2025.1599010 (PMC12425889; doi:10.3389/fbioe.2025.1599010)
Supplement: Supplementary file 1 [file DataSheet1.pdf]

1 Appendix A: Global TPS-RBF Interpolation Results Across Three Cases

A global TPS-RBF interpolation method was employed to evaluate the performance across three cases, which infers the positions of free internal nodes based on the known coordinates of constrained nodes. Specifically, the corresponding coordinates of constrained nodes in both the original and target configurations are extracted. For each free node, a fixed number $k(k = 100)$ of nearest constrained nodes are identified using a KD-tree search. A local TPS-RBF interpolator is then constructed based on these reference nodes, mapping the coordinates of the remaining free nodes from the original to the target configuration. The results show that the positions of some nodes exhibit noticeable distortions, as illustrated in Figure 1. Because the Laplace solver directly computes the coordinates of all free nodes in the presented framework, it is important to note that we did not employ a modular RBF interpolation in this study. The mesh quality after RBF interpolation for the three cases are summarized in Table 1.

Table 1 Global RBF interpolation results for three cases.

Quality criterion (number of elements)	CASE 1	CASE 2	CASE 3
$J < 0.3$	3500	10208	125
$J < 0$	1855	5683	38
Aspect ratio > 10	9203	7213	165

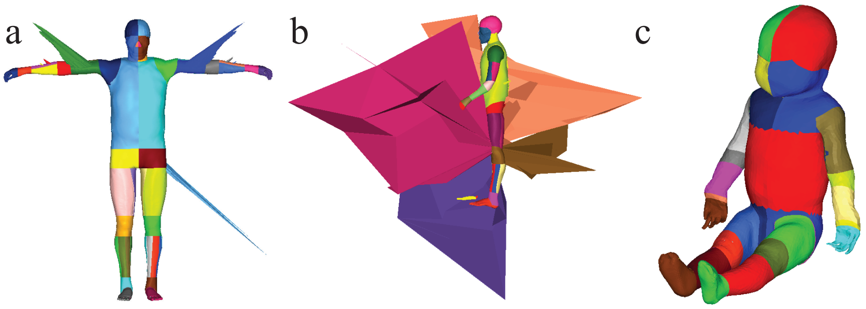

Fig. 1 HBM positioning results by global RBF integration using the same constraint nodes.
